# The management of unused and expired medications in Thai households: Influencing factors and prevailing practices

**DOI:** 10.1371/journal.pone.0309266

**Published:** 2024-08-27

**Authors:** Patranit Srijuntrapun, Kusawadee Maluangnon

**Affiliations:** 1 Department of Education, Faculty of Social Sciences and Humanities, Mahidol University, Salaya, Thailand; 2 Department of Pharmaceutical Care, Faculty of Pharmacy, Thammasat University, Pathum Thani, Thailand; Bina Nusantara University, INDONESIA

## Abstract

**Objective:**

Although the improper disposal of unused and expired medications represents a global environmental concern, its significance is often underestimated. This research delves into the practices and factors related to the management of unused and expired medications.

**Methods:**

Data was collected through a comprehensive survey of Thai households, with 400 structured questionnaires. Multiple regression was then employed to analyze the data.

**Results:**

Findings show an intriguing paradox. The participants in the survey demonstrated commendable knowledge regarding the appropriate management of unused or expired medications. Yet, this knowledge was not sufficiently put into practice. This study identifies the factors that have a positive influence on proper medication disposal practices, such as age, level of education, attitude, and perception. Surveyed participants exhibited a tendency to retain unused medications and discard expired ones with household waste. Most of them showed a low degree of medication management, pointing to the need for targeted interventions.

**Conclusion:**

This research provides tangible benefits for policymakers, healthcare practitioners, and researchers addressing waste management paradigms. In terms of policy, the study highlights the need for comprehensive interventions, including medication take-back systems, infrastructure development, and educational initiatives. Policymakers are urged to bridge the gap between knowledge and behavior by promoting access to collection points for proper medication disposal to avoid perpetuating the cycle of suboptimal medication management. This research informs strategies to encourage responsible medication management practices globally, addressing the adverse environmental and health impacts associated with the improper accumulation of medications.

## Introduction

Research on pharmaceutical household waste is crucial for addressing the intersection of public health and sustainable development goals (SDGs). The accumulation of unused or expired pharmaceuticals by households offers no therapeutic benefits and poses significant risks to human health and the environment when these medicines are improperly discarded. Proper medication disposal is key to achieving SDG12 (Responsible Consumption and Production) by preventing harm on ecosystems from pharmaceutical compounds [1]. It is also key to ensure that people have relevant information and awareness about sustainable development. Improper disposal of medication can lead to a wide range of risks [2]. Flushing medications or throwing them in the trash can introduce pharmaceutical compounds directly into the water supply, posing a threat to ecosystems and potentially contaminating drinking water sources, leading to environmental harm and potential health consequences [3], including antimicrobial resistance [4].

This issue has attracted the attention of global agencies such as the World Health Organization (WHO), which has developed practical guidelines for waste management in healthcare, including the disposal of unused or expired medications [5]. The practices for disposing of unused and expired medications vary significantly by country. These variations stem from differences in healthcare infrastructure and regulatory frameworks, all of which influence the behavior of households. High-income countries are often leaders in the implementation of comprehensive medication disposal systems. In these countries, there are widely accepted medication return programs organized by pharmacies, hospitals, or government agencies, offering accessible and environmentally friendly disposal methods to individuals. Examples of such countries include USA [6], Canada [7], Australia [8], United Kingdom [9], and New Zealand [10]. Even in these countries, however, there are challenges related to the methods of prescribing, dispensing, and storing medications at pharmacies. Additionally, legal considerations can undermine the implementation of waste reduction programs [11].

In developing countries, on the other hand, sufficiently comprehensive practices and regulations for the disposal of expired or unused medications are still lacking [12]. In low- and middle-income countries, the challenges are often related to the public systems of medication management, including inventory control and enforcement of regulations for pharmaceutical disposal. These problems often contribute to the accumulation of medicines [13, 14]. Despite the legal frameworks that regulate the disposal of hazardous and healthcare waste from hospitals and manufacturing facilities, these countries often lack clear policies for the safe disposal of medications by households [15]. Even where Medication Return Programs (MRPs) exist, they are often not mandatory. In Malaysia, for example, the MRP allows citizens to return unused medications to pharmacy counters at government hospitals for safe disposal. However, private pharmacies are not required to participate in the program [16]. As a result, most private pharmacies do not accept returned medications. This makes it difficult for the public to access appropriate disposal methods and ultimately leads to the improper disposal of medicines as household waste.

Several key factors influence how households dispose of unused medications, including knowledge of the potential dangers involved in their improper disposal [17] as well as limited access to disposal options. Inadequate infrastructure constitutes a significant barrier preventing households from participating in the disposal of waste medication [18]. However, minor improvements in disposal options, including access to suitable disposal locations and availability of bins, can have a significant impact on behaviors [19, 20]. Economic factors, such as financial costs, also have an influence on the recycling behavior of households [21]. Since people are primarily motivated by costs and benefits [22], financial incentives can both positively and negatively affect behavior [23]. Regulatory frameworks and their enforcement are of paramount importance in guiding household waste disposal practices. In countries with strict regulations and strong law enforcement, households tend to adhere to appropriate disposal practices.

Despite the recognized risks of improper medication disposal, there is still a significant research gap in understanding the scope of the problem and identifying effective solutions. This is due to limited data on inappropriate medication disposal practices among the general population and healthcare providers. The factors influencing these practices, including knowledge gaps, access to disposal facilities, and cultural attitudes, have been studied to some extent in some developing countries, such as Ethiopia [24–26], Zambia [27], Nepal [28], and Lebanon [29, 30]. However, this topic has not yet been studied in Thailand. Closing this research gap is crucial for the development of interventions and policies that target and promote appropriate medication disposal practices among the Thai population. The literature suggests that there are several variables that can be useful in predicting practices related to the management of unused or expired medications.

This study aims to narrow this knowledge gap by surveying behaviors related to the management of unused or expired medications in Thailand. It also aims to investigate the factors influencing such behaviors. This quantitative research study provides beneficial insight for organizations and researchers seeking to improve the management of unused and expired medications. From this work, we recommend policies to promote sustainable medication management practices, with the goals of aligning waste management practices with community lifestyles and fostering sustainable development.

## Methodology

### Description of the study area

Located in the central region of Thailand, the Phra Nakhon Si Ayutthaya municipality was selected for the study because it was recognized as a UNESCO world cultural heritage site in 1991 [31] and remains a popular destination, receiving with high travel flow through region. Another key reason is that in 2016 the Pollution Control Department ranked Phra Nakhon Si Ayutthaya as one of the provinces with the highest residual waste in Thailand [32]. Therefore, the region may be exposed to environmental contamination due to improper disposal of unused and expired medications.

The Phra Nakhon Si Ayutthaya municipality plays a pivotal role in the province’s waste management system. With a total population of 47,747 (22,701 males and 25,046 females) [33], the municipality is responsible for the maintenance of public order and the cleanliness of public areas. In addition, it takes care of waste disposal and sanitation. The region has one hospital and five comprehensive medical centers organized as primary care clusters (PCCs) that provide holistic healthcare services to the population. Furthermore, community health volunteers (CHVs) selected from local communities serve as change agents in promoting healthy behaviors and disseminating public health information. These individuals are instrumental in addressing the proper disposal of unused and expired medications within the community, making a significant contribution to the crucial task of pharmaceutical waste management in the region.

According to 2023 data from the National Health Security Office (NHSO), a total of 47 million individuals in Thailand were eligible to receive the National Health Insurance or Gold Card under the auspices of the NHSO. This program serves as the principal national health insurance mechanism, granting beneficiaries access to medications without incurring any financial burden [34]. Over the years, Thailand’s expenditure on pharmaceuticals has shown a consistent upward trend, growing from 86.54 billion baht ($2.32 billion) in 2008 to 180.58 billion baht ($4.85 billion) in 2018. A comparison with nations across the Asia-Pacific region reveals that Thailand’s per capita spending on medications is high, similar to developed countries. Such a disparity suggests that individuals enjoying quick and seamless access to healthcare services may be more likely to use medication inappropriately, which may result in a notable surplus of unused and expired pharmaceuticals [35].

### Data collection and characteristics of participants

This study employed a quantitative approach to collect data from households located in the Phra Nakhon Si Ayutthaya municipality during the period from 1 July to 30 September 2019. Data was obtained through face-to-face interviews using a questionnaire distributed to a representative sample of households in this area. According to 2019 statistics, there were 19,951 households in the Phra Nakhon Si Ayutthaya municipality. The minimum sample size for the study was determined to be 392 households using Taro Yamane’s formula with a confidence level of 95%. This level is suitable for a survey, particularly one that targets a finite population and employs simple random sampling methods [36]. To expedite the analysis of the data and the assessment of the results, the researchers decided to employ a comprehensive sample size of 400. This number surpasses the established minimum threshold sample and ensures a robust dataset, enhancing the statistical validity of the study’s findings. The participants were selected through convenience sampling from households that receive medication from hospitals, medical centers, and pharmacies in the area. Interviews continued until a total of 400 sets were obtained.

### Instruments

The collection and compilation of data was conducted through a questionnaire, which included the following sections: (1) general information of the sample group, including demographic details of respondents such as gender, educational attainment, and household income; (2) knowledge of unused or expired medications and their environmental impact, using 10 dichotomous questions gauging participants’ understanding; (3) attitudes regarding the management of unused or expired medications, evaluated through a 10-question survey with a 1 to 5 scale (1 = “strongly disagree”, 5 = “strongly agree”); (4) self-perception of efficacy in managing unused or expired medications, evaluated through a 5-question survey with a 1 to 5 scale (1 = “least confident”, 5 = “very confident”); (5) intrinsic motivations in managing unused or expired medications, evaluated through a 5-question survey with a 1 to 5 scale (1 = “least”, 5 = “highest”); (6) practices regarding the management of unused or expired medications, evaluated through a 7-question survey with a 1 to 3 scale (1 = “never”, 3 = “all the time”); and (7) issues regarding the methods employed to manage unused or expired medications.

The questionnaire underwent a two-step testing process. First, it was developed and reviewed by three experts to verify and refine the accuracy and suitability of its content prior to collecting the data. Then, a Reliability Test (pilot) was conducted with a sample of 30 questionnaire sets. The questionnaire’s reliability was assessed at a 95% confidence level using Cronbach’s alpha coefficient, which is a commonly employed measure of reliability. The formula for Cronbach’s alpha is α = (k * Σr) / (k—1 + Σr), where k is the number of items, Σr is the sum of item correlations, and α is a coefficient ranging from 0 to 1. In the social sciences, an alpha value between 0.7 and 0.8 is considered acceptable [37]. Results yielded a value of 0.802, underscoring the instrument’s credibility and its suitability for the present study.

### Data analysis

The data was analyzed using the SPSS software package (Statistical Package for the Social Sciences). The analysis included a synthesis of respondents’ demographic characteristics through descriptive statistics. The data was presented in terms of both frequency and percentage distributions. Additionally, key statistical measures such as mean (average) and standard deviation were calculated.

To assess the correlation between variables, a multiple regression analysis was employed. This investigation considered various factors, including knowledge, attitudes, self-perception of efficacy, intrinsic motivations, and practices regarding the management of unused or expired medications. All statistical analyses were performed using SPSS version 20.0 (IBM Corporation). A P-value lower than .05 was considered statistically significant.

### Ethical statement

The Ethics Committee of Mahidol University reviewed and approved the research project, with reference number 2019/133.0406. Prior to data collection, participants received information about the study’s objectives and their rights to participate or decline participation. Participants were also informed of the confidentiality of their data and were assured that the information collected would only be used for research purposes. Before taking part in the study, all participants signed a consent form. The study only included adult participants (aged above 18 years).

## Results

The study analyzed a representative sample of 400 individuals, including 222 males (55.5%) and 178 females (44.5%). 23.5% of participants were in the 41–50 year age range, closely followed by participants aged between 21 and 30 years (23.2%). Notably, 31.7% of participants held academic qualifications equivalent to a diploma or vocational certificate, while 28.5% of them had a bachelor’s degree. In terms of occupation, 44.7% of participants were civil servants or regular employees, while 22.0% of them were engaged in trade-related activities. Regarding income distribution, 53.4% of respondents reported monthly household incomes ranging between 10,000 and 30,000 Baht ($269-$806), while 42.7% indicated monthly incomes under 10,000 Baht ($269).

### Knowledge of unused or expired medications and their environmental impact

The survey included a set of yes/no questions designed to evaluate the knowledge and understanding of participants regarding the management of unused or expired medications and their potential environmental impact. Most respondents showed accurate comprehension of the environmental consequences of improperly discarding unused and expired medications, with 90.8% of them acknowledging that these medications, when improperly discarded and buried in municipal waste, could lead to detrimental effects on the environment. Furthermore, 85.5% of respondents were aware that the appropriate disposal method for unused or expired medications involved incineration. In addition, 74.7% of them correctly recognized that deteriorated and expired medications are categorized as hazardous waste. Nevertheless, a notable proportion of respondents (52.7%) incorrectly thought that unused and expired medications could be discarded alongside household waste (Table 1). The reliability of these items was acceptable (α = .701).

**Table 1:** Knowledge of unused or expired medications and their environmental impact.

Knowledge question	True (*n*, %)	False (*n*, %)
Eye drops containing antimicrobial agents to inhibit the growth of pathogens have a shelf life of no more than 1 month after being opened for use.	353 (88.2)	47 (11.8)
“Mfd. Date” or “MFE” stands for expiration date.	397 (99.3)	3(0.7)
Suspended gelatinous medications that clump together into solid lumps and do not disperse when shaken cannot be ingested anymore.	65 (16.3)	335 (83.7)
EXP 27/03/2018 means that the medication expired on March 27, 2018.	391 (97.7)	9 (2.3)
If a pill has changed color and has small black spots, it is still considered safe to consume.	362 (90.5)	38 (9.5)
Expired and deteriorated medications are considered hazardous waste and must be discarded only in the designated green bin.	299 (74.7)	101 (25.3)
Community management of unused and expired medications is responsibility of village health volunteers.	230 (57.5)	170 (42.5)
Unused and expired medications can be discarded with regular household waste.	189 (47.3)	211 (52.7)
Expired and unused medications discarded with municipal waste can dissolve and seep into rivers and canals.	237 (90.8)	163 (9.2)
Disposal of expired and unused medications must be done by incineration at temperatures ranging between 850 and 1,600 degrees Celsius.	342 (85.5)	58 (14.5)
Cronbach alpha = .701		

n = 400

Most participants (72.3%) exhibited a high level of knowledge (scores ranging from 6.7 to 10), while 27.2% of them showed a moderate level of knowledge (scores ranging from 3.3 to 6.7). Only a small minority (0.5%) demonstrated a low level of knowledge (scores ranging from 0 to 3.3).

### Attitudes regarding responsibility in managing unused or expired medications

Most participants agreed (60.0%) or strongly agreed (24.3%) with the notion that segregating unused and expired medications before disposing of them was beneficial to the environment. In addition, many participants agreed (53.8%) or strongly agreed (17.7%) that segregating medications showed social responsibility and contributed to mitigating the impact of waste on the environment. However, many respondents (48.5%) also believed that disposing of low quantities of unused and expired medications did not have a significant effect on the environment (Table 2). The reliability of these items was acceptable (α = .712).

**Table 2:** Attitudes regarding responsibility in managing unused or expired medications.

Item selected	Strongly agree	Agree	Not sure	Disagree	Strongly disagree	$\left(\bar{x}\right)$	Interpretation
Separating unused and expired medications before disposal helps to preserve the environment.	97 (24.3)	240 (60.0)	59 (14.7)	4 (1.0)	0 (0.0)	4.08	Positive
It is advisable to keep unused medications for potential future use.	5 (1.3)	25 (6.2)	45 (11.2)	312 (78.0)	13 (3.3)	2.24	Moderate
It is advisable to give unused medications to donation programs.	10 (2.5)	208 (52.0)	95 (23.7)	86 (21.5)	1 (0.3)	3.35	Moderate
There is no need to worry too much: everyone is affected by the environmental impact of unused and expired medications.	28 (7.0)	109 (27.2)	182 (45.5)	80 (20.0)	1 (0.3)	3.21	Moderate
Separating and disposing of unused and expired medications in the correct manner can be a complicated task.	70 (17.5)	130 (32.5)	62 (15.5)	136 (34.0)	2 (0.5)	3.33	Moderate
There is no need to segregate unused and expired medications, given that waste collectors will ultimately mix all the waste together.	27 (6.7)	125 (31.3)	136 (34.0)	110 (27.5)	2 (0.5)	3.16	Moderate
Disposing of unused and expired medications is responsibility of the municipality, not of the public.	0 (0.0)	165 (41.2)	126 (31.5)	108 (27.0)	1 (0.3)	3.14	Moderate
Separating and disposing of unused and expired medications demonstrates social responsibility and helps to reduce the impact of waste.	71 (17.7)	215 (53.8)	96 (24.0)	16 (4.0)	2 (0.5)	3.84	Positive
Since villagers pay for waste collection, it is not necessary to segregate waste.	31 (7.7)	172 (43.0)	130 (32.5)	64 (16.0)	3 (0.8)	3.41	Moderate
Discarding unused and expired medications in small quantities is unlikely to have a significant impact on the environment.	3 (0.8)	194 (48.5)	164 (41.0)	37 (9.2)	2 (0.5)	3.40	Moderate
Cronbach alpha = .712							
**Total**						**3.31**	Moderate

n = 400

The study shows that 96.7% of respondents had a positive attitude (scores ranging from 27 to 50), 3.3% of them had a neutral attitude (14 to 26), while none of them had a negative attitude (0 to 13).

### Self-perceptions regarding efficacy in managing unused or expired medications

The survey explored the self-perception of participants regarding their efficacy in managing unused or expired medications. A large proportion of respondents expressed confidence (53.0%) or high confidence (19.2%) in their ability to donate unused medications to others. Conversely, 56.0% of participants displayed less confidence in their ability to find accurate information about the segregation and identification of expired medications. Moreover, 58.0% of respondents were not so confident in their ability to accurately locate disposal points for unused or expired medications. Participants reported moderate confidence (51.5%) or low confidence (29.3%) in their ability to reduce the environmental impacts caused by unused or expired medications (Table 3). The reliability of these items was acceptable (α = .875).

**Table 3:** Self-perceptions regarding efficacy in managing unused or expired medications.

Item	Least Confident	Not so confident	Moderately confident	Confident	Very confident	$\left(\bar{x}\right)$	Interpretation
Ability to find information about segregation and identification of expired medications.	20 (5.0)	224 (56.0)	81 (20.3)	51 (12.7)	24 (6.0)	2.59	Moderately confident
Ability to find proper disposal sites for unused or expired medications.	37 (9.3)	232 (58.0)	64 (16.0)	67 (16.7)	0 (0.0)	2.40	Moderately confident
Ability to segregate expired medications.	15 (3.7)	97 (24.3)	206 (51.5)	60 (15.0)	22 (5.5)	2.94	Moderately confident
Ability to donate unused medications to others.	0 (0.0)	9 (2.3)	102 (25.5)	212 (53.0)	77 (19.2)	2.89	Moderately confident
Ability to reduce the environmental impact caused by unused or expired medications.	18 (4.5)	117 (29.3)	206 (51.5)	37 (9.2)	22 (5.5)	2.82	Not so confident
Cronbach alpha = .875							
		**Total**				**2.72**	Moderately confident

n = 400

Most respondents (81.0%) exhibited a moderate level of confidence in their efficacy (scores ranging between 9 and 16). A smaller proportion (14.5%) displayed a high level of confidence (scores ranging between 17 and 25). Lastly, only 4.5% of respondents expressed low levels of confidence in their efficacy (scores ranging between 0 and 8).

### Intrinsic motivations for managing unused or expired medications

The survey showed that participants had significant intrinsic motivations for segregating unused or expired medications. Notably, 40.0% of respondents were highly motivated by the prospect of contributing to environmental preservation in general, 38.5% by the belief that these medications could aid underprivileged individuals in need of medical assistance, and 38.3% by the opportunity to safeguard public health against the adverse effects of these medications. On the other hand, participants were moderately motivated by the opportunity to assist in reducing the country’s financial burden of purchasing additional medications (52.5%) or lowering the costs associated with hazardous waste disposal by government institutions and hospitals (50.0%) (Table 4). The reliability of these items was acceptable (α = .801).

**Table 4:** Levels of intrinsic motivation for managing unused or expired medications.

Intrinsic Motivation	Highest	High	Moderate	Low	Least	$\left(\bar{x}\right)$	Interpretation
Reducing the financial burden of purchasing additional medicines by the government.	7 (1.7)	82 (20.5)	210 (52.5)	95 (23.8)	6 (1.5)	2.97	Moderate
Reducing the expenses related to the disposal of hazardous waste by the government or public hospitals.	1 (0.3)	107 (26.7)	200 (50.0)	92 (23.0)	0 (0.0)	3.04	Moderate
Donating to help underprivileged sick people.	40 (10.0)	154 (38.5)	80 (20.0)	95 (23.8)	31 (7.7)	3.19	Moderate
Protecting people’s health from the hazards of discarded drugs.	13 (3.3)	153 (38.3)	142 (35.4)	77 (19.3)	15 (3.7)	3.18	Moderate
Contributing to preserve the environment.	23 (5.7)	162 (40.5)	117 (29.3)	69 (17.3)	29 (7.2)	3.20	Moderate
Cronbach alpha = .801							
**Total**						**3.11**	Moderate

n = 400

Most respondents (48.5%) showed a moderate level of intrinsic motivation for managing unused or expired medications (scores ranging between 9 and 16). A significant proportion (46.0%) exhibited a high level of intrinsic motivation (scores ranging between 17 and 25). A smaller subset of respondents (5.5%) seemed to have low levels of intrinsic motivation (scores ranging between 0 and 8).

### Management of unused or expired medications

In this paper, transferring medications to others is considered as incorrect management of unused medications, as it could lead to potential health risks and side effects. Instead, the recommendation is to donate unused medications to hospitals or to return them to unwanted medication programs. In the case of expired medications, dropping them off at designated collection points in hospitals or community health care centers is considered as proper disposal, while throwing them away with household waste is improper disposal.

The survey showed that participants gave away unused medications to relatives or friends who suffered similar illnesses on some occasions (72.0%) or all the time (21.7%). On the other hand, 78.4% of respondents had never donated unused medications to specific programs intended for this purpose. A significant proportion of respondents (78.0%) indicated that they never discarded unused or expired medications at hospital disposal points. An even more substantial proportion (91.0%) never gave such medications to village health volunteers for proper management. Notably, 53.7% of respondents reported that they occasionally disposed of unused or expired medications in the red waste bins (hazardous waste bins) available in their communities. Additionally, 57.0% of respondents acknowledged disposing of unused or expired medications together with other household waste (Table 5). The reliability of these items was acceptable (α = .794).

**Table 5:** Management of unused or expired medications.

Practice	All the time	Sometimes	Never	$\left(\bar{x}\right)$	Interpretation
Gives unused medications to relatives or friends who suffer similar illnesses.	87 (21.7)	288 (72.0)	25 (6.3)	2.16	Moderate
Keeps unused medications in case of recurring sickness in the future.	6 (1.5)	191 (47.7)	203 (50.7)	1.51	Low
Disposes of unused and expired medications together with household waste.	30 (7.5)	228 (57.0)	142 (35.5)	1.72	Moderate
Gives unused medications to drug donation programs.	1 (0.3)	85 (21.3)	314 (78.4)	1.22	Low
Gives unused medications to village health volunteers for ongoing management.	0 (0.0)	36 (9.0)	364 (91.0)	1.09	Low
Discards unused medications at the designated disposal points in the hospital.	0 (0.0)	88 (22.0)	312 (78.0)	1.22	Low
Discards unused medications in the community’s red waste bin.	0 (0.0)	215 (53.7)	185 (46.3)	1.54	Low
Cronbach alpha = .794					
**Total**				**1.49**	**Low**

n = 400

The vast majority of respondents (95.0%) had low scores (ranging from 0 to 7) in terms of their management of unused or expired medications. The rest of participants showed moderate (2.5% scored between 8 and 14) or high levels of management (2.5% scored between 15 and 21).

### Factors affecting the management of unused or expired medications

In order to determine the factors contributing to the management of unused or expired medications, the assumptions for multiple linear regression analysis, including normality of distribution, linearity, and independence of outcome variables, were validated. The β-value (regression coefficient) was used to verify if the independent variables had explanatory power. An analysis of the relationships among the variables was then carried out (Table 6). It was found that the knowledge score had a negative correlation with the self-perception score (r = -.125). In addition, the attitude score had a positive correlation with the perception score (r = .568) as well as a positive correlation with the management practices score (r = .405). Finally, the self-perception score had a significant positive correlation with the management practices score (r = .390).

**Table 6:** Results of the analysis of interrelationships between variables.

	Total knowledge	Total attitude score	Total self-perception of efficacy score	Total intrinsic motivation score	Total management practices score
Total knowledge score	1.000	-0.088	-0.125*	-0.025	-0.041
Total attitude score	-0.088	1.000	0.568**	0.059	0.405**
Total self-perception of efficacy score	-0.125*	0.568**	1.000	-0.066	0.390**
Total intrinsic motivation score	-0.025	0.059	-0.066	1.000	-0.086
Total management practices score	-0.041	0.405**	0.390**	-0.086	1.000

*Significance level of 0.05

**Significance level of 0.01

To complement this analysis, a multiple regression was conducted on the relationships between the variables influencing the management of unused or expired medications, namely knowledge, attitudes, self-perception of efficacy, intrinsic motivation, and management practices. The results are presented in Table 7.

**Table 7:** Results of the multiple regression analysis between independent variables influencing the management of unused or expired medications.

Factor	Beta	Std. Error	t	p-value
Gender	-0.109	0.174	-0.627	0.531
Age	0.021**	0.007	3.055	0.002
Academic qualifications				
No education (reference group)				
Primary education	1.425	0.755	1.888	0.060
Lower secondary education	1.296	0.720	1.800	0.073
Upper secondary education	1.230	0.696	1.766	0.078
Diploma or vocational certificate	1.579*	0.694	2.276	0.023
Bachelor’s degree	1.743*	0.706	2.468	0.014
Occupation				
No occupation (reference group)				
Civil servant or regular employee	-0.028	0.299	-0.095	0.924
Trade-related activity	0.329	0.291	1.130	0.259
Daily basis work	0.255	0.324	0.787	0.432
Other	-0.494	1.223	-0.404	0.686
Income				
Less than 10,000 baht (less than $269) (reference group)				
10,000-30,000 baht ($269-$806)	0.080	0.225	0.355	0.723
More than 30,000 baht (more than $806)	0.334	0.514	0.650	0.516
Total knowledge score	-0.144*	0.062	-2.315	0.021
Total attitude score	0.131***	0.024	5.433	0.000
Total self-perception of efficacy score	0.105**	0.031	3.444	0.001
Total intrinsic motivation score	-0.053*	0.024	-2.173	0.030
R^2^	0.279			
Adjusted R^2^	0.247			

Note:

*p<0.05,

**p<0.01,

***p<0.001

This study provides some insights into the factors impacting the management of unused or expired medications. Notably, gender does not seem to exert a discernible influence on such practices. However, age emerges as a significant determinant, with a positive association (beta = 0.021).

Educational attainment is another influential factor in the reported use of used and expired medications. The scores of participants possessing a degree or diploma exceeded their non-educated counterparts by 1.579 points. Similarly, participants with a bachelor’s degree obtained scores 1.743 points higher than participants with no education.

Regarding the knowledge variable, a salient finding of the study is that knowledge scores seemed to be associated with behaviors linked to the disposal and categorization of unused or expired medications. For each incremental point in the knowledge score, there was a corresponding decrease of 0.144 points in the management score connected to the proper handling of these medications. Similarly, the significant beta value of -0.053 in the motivation variable shows that motivation also has an influence on behaviors associated with the disposal and segregation of unused or expired medications.

Furthermore, the attitude variable (beta = 0.131) and the self-perception of efficacy variable (beta = 0.105) also emerged as relevant factors. Considering the coefficient of determination (r^2^), the cumulative effect of all the independent variables in the equation explains 27.9% of the variance in the observed changes in the variable measuring the management of unused or expired medications. The statistical error of 72.1% in the regression model can be attributed to factors not captured in the variables considered in the regression.

### Causes underlying the generation of unused or expired medications

We observed that 47.9% of respondents stopped taking their medications on their own, 26.1% obtained additional medications beyond the prescribed ones, and 21.3% did not follow doctors’ prescriptions. The details of other contributing factors are presented in S1 Table.

The study also showed that the causes behind the generation of expired medications were different from those behind the generation of unused medications. In 30.6% of the cases, medications expired due to inadequate storage, leading to their deterioration. The purchase of excess medication (18.9%) and the discontinuation of medication by the patients (18.3%) were also prevalent causes of expiration. Other factors contributing to expiration were the inadequate monitoring of medications remaining at home (13.8%) and the failure to adhere to instructions given by doctors (12.3%). Only a small percentage of expirations (6.1%) were related to patients’ passing away (see S2 Table).

The study also showed that the reasons given for generating expired medications were different from the reasons given for generating unused medications. Unused medications mainly arose from not adhering to doctors’ instructions, while expired medications were mostly due to a lack of proper knowledge and understanding of the appropriate methods of storage.

### Methods of managing unused or expired medications

Understanding the methods employed to manage unused or expired medications is crucial in mitigating instances of inappropriate disposal. Most respondents (86.0%) simply stored them, followed by those who shared them with others (6.7%). A small percentage of respondents (4.8%) discarded them, while a mere 2% of respondents reported returning them to healthcare facilities (see S3 Table). Notably, the predominant approach (73.7%) for the disposal of unused medications involved placing them in household trash bins, followed by community trash bins (26.3%).

The way households manage expired medications differs from the way they manage unused ones. Based on the study, 60.5% of respondents discarded expired medications, followed by those who stored them (25.7%), dropped them off at hospitals or healthcare facilities (12.3%), or followed other methods (1.5%) (see S4 Table). The study also asked about the locations where people discarded expired medications. 38.4% of respondents used household trash bins, 31.4% community trash bins, and 24.4% red community trash bins.

Based on the survey, it seems that most households keep unused medications at home, either for their own use in the future or to give them to others. On the other hand, those who dispose of expired medications believe they will not be beneficial in the future. In any case, the management of both unused and expired medications generally involves inappropriate disposal methods with potentially harmful environmental impacts.

## Discussion

### Presence and management of unused or expired medications in households

The study found that unused and expired medications are kept in households for different reasons. Unused medications are mainly found in households due to people not following doctors’ instructions. Based on the survey, 47.9% of respondents stopped taking their medication before completing the course, 26.1% used additional self-sourced medications alongside the prescribed ones, and 21.3% did not follow the prescription given by the doctor (S1 Table). A similar situation is described in a study conducted in southwest Ethiopia, where 56.7% of patients sought to improve their own medical condition [26].

On the other hand, the accumulation of expired medications by the surveyed households was mainly due to inadequate storage (30.6%), purchase or prescription of excessive medication (18.9%), discontinuation of medication by the patients (18.3%), or lack of monitoring of medications remaining at home (13.8%) (S2 Table). These reasons reveal a lack of knowledge regarding the proper use and storage of medications on the part of respondents. Similar results were found by researchers working in the eastern region of Saudi Arabia [38]. Additionally, this situation is reminiscent of countries like Lebanon where medications are easily accessible without prescription [39]. This can lead to adverse drug reactions, as well as contributing to the accumulation of unused and expired medications.

The study also identified different methods employed by households for managing unused or expired medications. Most respondents (86.0%) kept unused medications at home without taking any action (S3 Table). In addition, many of them (60.5%) discarded expired medications together with household waste (S4 Table). Similar behaviors have been identified in other countries. For instance, in Lebanon, 60.3% of individuals stored unused medications at home until they expired, while 70.9% tended to discard expired medications [29]. In Zambia, 95.0% of individuals stored unused medications at home until expiration and 97% discarded them after they had expired [27]. In Nepal, 37.1% of individuals stored unused medications and 64.3% discarded expired medications [28]. In several other countries, however, the predominant practice was to dispose of both unused and expired medications in regular waste bins. For instance, in the community of Adigrat City in northern Ethiopia, 63.0% of respondents discarded unused medications in such a way while 75.2% did the same thing with expired medications [25]. In Indonesia, 64.4% of people discarded unused medications and 82.1% discarded expired medications [40].

### Factors affecting the management of unused or expired medications

This study shows that most people have a good knowledge about unused or expired medications and their environmental impact. However, 52.7% of respondents incorrectly believed that such medications could be discarded along with household waste (Table 1). This discrepancy can be attributed to the lack of well-established medication take-back systems in Thailand, both at pharmacies and healthcare facilities. The lack of dedicated waste disposal bins in communities further restricts the options for individuals. In such circumstances, people have limited alternatives and the convenience of discarding medications in a familiar environment becomes the default choice. This leads individuals to dispose of medications together with regular household waste as a matter of habit over extended periods.

Despite their misconceptions regarding disposal methods, most respondents expressed a positive attitude (24.3% strongly agreed and 60.0% agreed) towards separating and discarding medications in order to contribute to the preservation of the environment. This finding aligns with global trends, as evidenced by similar levels of support in Ethiopia (95.5%) [26] and Afghanistan (98.0%) [12]. This highlights the universal concern for the environmental and health implications of improper disposal of medications.

Nearly all (95%) of respondents reported poor practices in managing unused or expired medication (Table 5). This situation is reminiscent of studies conducted in India where respondents were aware of the dangers of improper medication disposal but still disposed of medications using inadequate methods [41, 42].

The multivariate linear regression analysis modeled self-report behavior in relation to the disposal and separation of unused or expired medications. This analysis revealed several notable associations. In particular, age (beta = 0.021) and educational level (having a degree or diploma: beta = 1.579; having a bachelor’s degree: beta = 1.743) appeared to be positively correlated with better practices in disposal and separation of medications. Attitude (beta = 0.131) and self-perception (beta = 0.105) were also linked to recommended practices. On the other hand, knowledge (beta = -0.144) and motivation (beta = -0.053) were associated with poor practices (Table 7). This may be due to the lack of designated waste bins for the disposal of unused or expired medications in the study area, coupled with challenges in accessing appropriate disposal facilities. This has led to the practice of discarding such medications in household waste bins. In this sense, the lack of necessary infrastructure seems to be one of the most significant barriers for households to safely dispose of their medications [25]. In such contexts, knowledge tends to be negatively correlated with behavior.

The primary intervention needed, therefore, is the implementation of essential infrastructure. It is particularly important to provide easily accessible collection points for the proper disposal of unused and expired medications. Even small changes in convenience can substantially influence behaviors, encouraging appropriate waste disposal and potentially cultivating new habits that align with sustainable environmental practices [25]. As has been observed in developed countries, these interventions have the potential to promote environmentally friendly behaviors and facilitate the return of medications.

In addition to developing medication return systems and the infrastructure of waste bins for their disposal, it is necessary to reduce the occurrence of unused and expired medications in households. This can be achieved by fostering greater knowledge and awareness among the public regarding the use and proper disposal of medications. Such efforts can lead to more responsible medication management practices within the community. Healthcare professionals and experts should provide guidance on appropriate medication storage and safe disposal of common household medications, thereby promoting responsible practices. This approach has been documented in Nepal [28] and southwest Ethiopia [26].

One limitation of this study is the potential for bias in the responses as participants tend to subjectively report their own behaviors, often under the influence of prevailing social values. For instance, participants may report that they dispose of unused and expired medications in an environmentally responsible manner, such as by returning them to designated hospital collection points when they actually discard them with regular household waste for convenience. Similarly, participants may inaccurately recall their adherence to proper storage practices or how frequently they refill medications. Therefore, future research should consider using mixed method approaches, including both surveys and qualitative data collection through interviews or group discussions, to gain a deeper understanding of participants’ perspectives and behaviors. This would help mitigate the biases on the perceived prevalence and effectiveness of the different medication management strategies. It would also prevent potential misinformation regarding policy and intervention efforts aimed at promoting safe medication practices.

### Research implications and conclusions

The findings of this research study have significant theoretical and policy implications for addressing the complex issue of unused and expired medications in households.

#### Theoretical implications

The study contributes to the theoretical understanding of medication management by highlighting how behavior, knowledge, and culture influences the accumulation of unused and expired medications by patients. The positive correlations observed underscore the importance of factors like age, educational level, and attitude in shaping the behaviors of individuals. This suggests that theoretical frameworks should consider socio-cultural and demographic characteristics when exploring medication management practices. In addition, the study highlights the potential tension between knowledge and behavior, emphasizing the importance of turning contextual barriers into positive practices by supporting the implementation of necessary infrastructure, such as bins for unused and expired medications.

#### Policy implications

This research underscores the urgent need for targeted policy interventions to mitigate the causes and consequences of medication wastage. The practice of storing unused medications at home and discarding them with regular household waste that have been identified in this study reveal the inadequacy of current disposal systems. Policymakers should prioritize the establishment of well-defined medication take-back systems at pharmacies and healthcare facilities. Moreover, the lack of designated waste disposal bins in local communities points to a critical infrastructure gap that requires policy attention. Governments and local authorities should invest in developing adequate infrastructure, such as easily accessible collection points, to facilitate proper disposal and reduce the environmental impact of medication waste.

Furthermore, this study highlights the need for educational initiatives targeting both the general public and healthcare professionals. Policies should focus on fostering awareness about the environmental and health implications of improper medication disposal. Implementing educational programs can bridge the gap between knowledge and behavior, encouraging responsible practices. Healthcare professionals should play a pivotal role in providing guidance on appropriate medication storage and disposal, in line with successful initiatives observed in countries like Nepal and Ethiopia. In addition, policymakers should address the access to medications without prescription, a problem which is also found for example in Lebanon. This issue requires regulatory measures to prevent adverse drug reactions and curb the accumulation of unused and expired medications.

In conclusion, this research suggests that effective policies must address behavioral, cultural, and infrastructural aspects. By considering these theoretical insights and policy recommendations, stakeholders can collaboratively work towards a comprehensive strategy to reduce medication waste and promote responsible medication management practices within communities.

## Supporting information

S1 File Original survey questionnaire used in the study. (DOCX)

S1 Table Causes behind the generation of unused medications. (DOCX)

S2 Table Causes behind the generation of expired medications. (DOCX)

S3 Table Methods of managing unused medications. (DOCX)

S4 Table Methods of managing expired medications. (DOCX)
