# Supplementary material for: The management of unused and expired medications in Thai households: Influencing factors and prevailing practices
Source: PLoS One. 2024 Aug 27;19(8):e0309266. doi: 10.1371/journal.pone.0309266 (PMC11349084; doi:10.1371/journal.pone.0309266)
Supplement: S1 File — (DOCX) [file pone.0309266.s001.docx]

**S1 File. Original survey questionnaire used in the study**

**Section 1** Demographic information

Gender ❒ Male ❒ Female

Age (years)

Academic qualifications ❒ not studied ❒ primary school ❒ Junior high school ❒ Junior high school

❒ diploma or vocational certificate ❒ bachelor's degrees ❒ master’s degrees ❒ doctor degrees

❒ other………………

Occupation ❒ not employed ❒ civil servants or regular employees ❒ trade-related activities

❒ daily wage worker ❒ other………………

Income ❒ <10,000 baht ❒ 10,000 – 30,000 baht ❒ > 30,000 baht

**Section 2** Knowledge of unused or expired medications and their environmental impact

| **Knowledge question** | **True (*n*, %)** | **False (*n*, %)** |
| --- | --- | --- |
| Eye drops containing antimicrobial agents to inhibit the growth of pathogens have a shelf life of no more than 1 month after being opened for use. |  |  |
| “Mfd. Date” or “MFE” stands for expiration date. |  |  |
| Suspended gelatinous medications that clump together into solid lumps and do not disperse when shaken cannot be ingested anymore. |  |  |
| EXP 27/03/2018 means that the medication expired on March 27, 2018. |  |  |
| If a pill has changed color and has small black spots, it is still considered safe to consume. |  |  |
| Expired and deteriorated medications are considered hazardous waste and must be discarded only in the designated green bin. |  |  |
| Managing unused and expired medications in the community is the responsibility of village health volunteers. |  |  |
| Unused and expired medications can be discarded with regular household waste. |  |  |
| Expired and unused medications discarded with municipal waste can dissolve and seep into rivers and canals. |  |  |
| Disposal of expired and unused medications must be done by incineration at temperatures ranging between 850 and 1,600 degrees Celsius. |  |  |

**Section 3** Attitudes regarding responsibility in managing unused or expired medications

| **Item selected** | **Strongly agree** | **Agree** | **Not sure** | **Disagree** | **Strongly disagree** |
| --- | --- | --- | --- | --- | --- |
| Separating unused and expired medications before disposal helps to preserve the environment. |  |  |  |  |  |
| It is advisable to keep unused medications for potential future use. |  |  |  |  |  |
| It is advisable to give unused medications to donation programs. |  |  |  |  |  |
| There is no need to worry too much: everyone is affected by the environmental impact of unused and expired medications. |  |  |  |  |  |
| Separating and disposing of unused and expired medications in the correct manner can be a complicated task. |  |  |  |  |  |
| There is no need to segregate unused and expired medications, given that waste collectors will ultimately mix all the waste together. |  |  |  |  |  |
| Disposing of unused and expired medications is responsibility of the municipality, not of the public. |  |  |  |  |  |
| Separating and disposing of unused and expired medications demonstrates social responsibility and helps to reduce the impact of waste. |  |  |  |  |  |
| Since villagers pay for waste collection, it is not necessary to segregate waste. |  |  |  |  |  |
| Discarding unused and expired medications in small quantities is unlikely to have a significant impact on the environment. |  |  |  |  |  |

**Section 4** Self-perceptions regarding efficacy in managing unused or expired medications

| **Item** | **Least Confident** | **Not so confident** | **Moderately confident** | **Confident** | **Very confident** |
| --- | --- | --- | --- | --- | --- |
| Ability to find information about segregation and identification of expired medications. |  |  |  |  |  |
| Ability to find proper disposal sites for unused or expired medications. |  |  |  |  |  |
| Ability to segregate expired medications. |  |  |  |  |  |
| Ability to donate unused medications to others. |  |  |  |  |  |
| Ability to reduce the environmental impact caused by unused or expired medications. |  |  |  |  |  |

**Section 5** Intrinsic motivations for managing unused or expired medications

| **Intrinsic Motivation** | **Highest** | **High** | **Moderate** | **Low** | **Least** |
| --- | --- | --- | --- | --- | --- |
| Reducing the financial burden of purchasing additional medicines by the government. |  |  |  |  |  |
| Reducing the expenses related to the disposal of hazardous waste by the government or public hospitals. |  |  |  |  |  |
| Donating to help underprivileged sick people. |  |  |  |  |  |
| Protecting people’s health from the hazards of discarded drugs. |  |  |  |  |  |
| Contributing to preserve the environment. |  |  |  |  |  |

**Section 6** Management of unused or expired medications

| **Practice** | **All the time** | **Sometimes** | **Never** |
| --- | --- | --- | --- |
| Distributes unused medications to relatives or friends who suffer similar illnesses. |  |  |  |
| Keeps unused medications in case of recurring sickness in the future. |  |  |  |
| Disposes of unused and expired medications together with household waste. |  |  |  |
| Gives unused medications to drug donation programs. |  |  |  |
| Gives unused medications to village health volunteers for ongoing management. |  |  |  |
| Discards unused medications at the designated disposal points in the hospital. |  |  |  |
| Discards unused medications in the community’s red waste bin. |  |  |  |

**Section 7** Causes underlying the generation of unused or expired medications

Causes for the generation of unused medications (choose more than one option)

1. ❒ Problems raised from medication use

2. ❒ Over-prescription by physicians

3. ❒ Changes in medication type/method of consumption by physicians

4. ❒ Did not follow doctors’ prescriptions

5. ❒ Obtained additional medications beyond the prescribed ones

6. ❒ Lack of fixed appointment dates

7. ❒ Stopped taking their medications on their own

8. ❒ Patient fatalities 9. ❒ other………………

Causes for the generation of expired medications (choose more than one option)

1. ❒ Improper storage leading to medication deterioration

2. ❒ Lack of inventory check for remaining medication at home

3. ❒ Receiving/purchasing excess medication unnecessarily

4. ❒ Non-adherence to physician's instructions

5. ❒ Self-discontinuation of medication

6. ❒ Patient fatalities 7. ❒ other………………

**Section 8** Methods of managing unused or expired medications

Methods of managing unused medications

1. ❒ Use leftover medications until finished before starting a new prescription as directed by the physician

2. ❒ Store medications without specific action

3. ❒ Return medications to healthcare facilities

4. ❒ Share medications with others

5. ❒ Donate medicine to unused medication donation program

6. ❒ Dispose of unused medications

7. ❒ other………………

Methods of managing expired medications

1. ❒ Store medications without specific action

2. ❒ Drop off medications at hospitals or healthcare facilities

3. ❒ Discard expired medications

4. ❒ other
