# Supplementary material for: The management of unused and expired medications in Thai households: Influencing factors and prevailing practices
Source: PLoS One. 2024 Aug 27;19(8):e0309266. doi: 10.1371/journal.pone.0309266 (PMC11349084; doi:10.1371/journal.pone.0309266)
Supplement: S1 Table — (DOCX) [file pone.0309266.s002.docx]

**S1 Table. Causes behind the generation of unused medications** (select more than one option)

n=574

| **Causes behind the generation of unused medications** | ***n*, %** |
| --- | --- |
| Problems derived from the use of the medication | 7 (1.2) |
| Over-prescription by physicians | 4 (0.7) |
| Changes in medication type/method of consumption by physicians | 5 (0.9) |
| Failure to follow doctors’ prescriptions | 122 (21.3) |
| Obtained additional medications beyond the prescribed ones | 150 (26.1) |
| Lack of fixed appointment dates | 6 (1.0) |
| Stopped taking their medications on their own | 275 (47.9) |
| Patient fatalities | 5 (0.9) |
