# Supplementary material for: The management of unused and expired medications in Thai households: Influencing factors and prevailing practices
Source: PLoS One. 2024 Aug 27;19(8):e0309266. doi: 10.1371/journal.pone.0309266 (PMC11349084; doi:10.1371/journal.pone.0309266)
Supplement: S2 Table — (DOCX) [file pone.0309266.s003.docx]

**S2 Table. Causes behind the generation of expired medications** (select more than one option)

n=628

| **Causes behind the generation of expired medications** | ***n*, %** |
| --- | --- |
| Improper storage leading to medication deterioration | 192 (30.6) |
| Failure to check inventory of medications remaining at home | 87 (13.8) |
| Unnecessary receipt or purchase of excess medication | 119 (18.9) |
| Non-adherence to physician's instructions | 77 (12.3) |
| Self-discontinuation of medication | 115 (18.3) |
| Patient fatalities | 38 (6.1) |
