# Supplementary material for: The management of unused and expired medications in Thai households: Influencing factors and prevailing practices
Source: PLoS One. 2024 Aug 27;19(8):e0309266. doi: 10.1371/journal.pone.0309266 (PMC11349084; doi:10.1371/journal.pone.0309266)
Supplement: S3 Table — (DOCX) [file pone.0309266.s004.docx]

**S3 Table.** **Methods of managing unused medications**

n=400

| **Methods of managing unused medications** | ***n*, %** |
| --- | --- |
| Used them until finished before starting a new prescription, as directed by the physician | 2 (0.5) |
| Stored them | 344 (86.0) |
| Returned them to healthcare facilities | 8 (2.0) |
| Shared them with others | 27 (6.7) |
| Gave them to a donation program | 0 (0) |
| Discarded them | 19 (4.8) |
