# Supplementary material for: The management of unused and expired medications in Thai households: Influencing factors and prevailing practices
Source: PLoS One. 2024 Aug 27;19(8):e0309266. doi: 10.1371/journal.pone.0309266 (PMC11349084; doi:10.1371/journal.pone.0309266)
Supplement: S4 Table — (DOCX) [file pone.0309266.s005.docx]

**S4 Table.** **Methods of managing expired medications**

n=400

| **Methods of managing expired medications** | ***n*, %** |
| --- | --- |
| Stored them without taking any action | 103 (25.7) |
| Dropped them off at hospitals or healthcare facilities | 49 (12.3) |
| Discarded them | 242 (60.5) |
| Other | 6 (1.5) |
